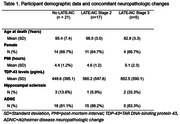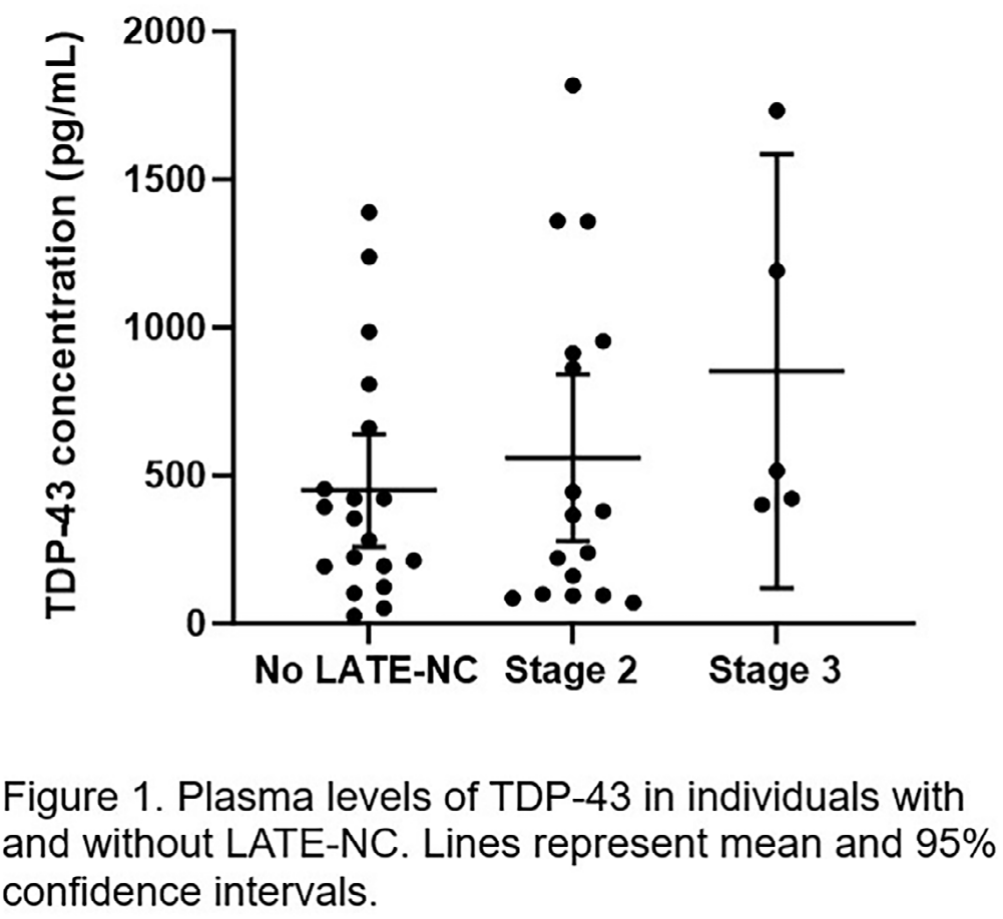# The potential of plasma TDP‐43 as a biomarker for limbic predominant age related TDP‐43 encephalopathy

**DOI:** 10.1002/alz.093180

**Published:** 2025-01-09

**Authors:** Lorena Sordo, Anne‐Marie C Leiby, Rond Malhas, James Barlow, Robert A. Rissman, Claudia H. Kawas, María M. M. Corrada, Elizabeth Head, S. Ahmad Sajjadi

**Affiliations:** ^1^ University of California Irvine, Irvine, CA USA; ^2^ University of California, Irvine, CA USA; ^3^ University of California, Irvine, Irvine, CA USA; ^4^ University of California San Diego, La Jolla, CA USA; ^5^ University of California, San Diego, La Jolla, CA USA

## Abstract

**Background:**

Despite recent advances in biomarkers discovery for neurodegenerative diseases, there are currently no in vivo biomarkers for limbic‐predominant age‐related TDP‐43 encephalopathy (LATE‐NC). A few studies have suggested that plasma TDP‐43 can be an in vivo marker of frontotemporal dementia due to TDP‐43 pathology and amyotrophic lateral sclerosis. In this pilot study, we aimed to assess plasma TDP‐43 concentrations as a biomarker in individuals with autopsy confirmed LATE‐NC.

**Methods:**

We measured plasma TDP‐43 levels in a convenience sample of 48 participants of The 90+ study (n=40) and UCI Alzheimer’s Disease Research Center (n=8) with available brain autopsy results. Of these, 26 (54.2%) were TDP‐43(+) [LATE stages 2 (n=21) and 3 (n=5)], and 22 (45.8%) were TDP‐43(‐) cases. Cases with LATE Stage 1 were not included in this study. TDP‐43 levels were measured using the Simoa TDP‐43 Advantage kit on the HD‐X platform following manufacturer’s protocol.

**Results:**

We were able to successfully measure TDP‐43 concentrations in all plasma samples. Samples with coefficient of variation (%CV) >20% were excluded from analysis (n=5). Participants ages ranged from 72 to 104 years old (mean=95.2, SD=7.3). Of these, 29 (67.4%) were female. Demographics and neuropathological characteristics stratified by LATE‐NC presence and stage are depicted in table 1. Although non‐parametric tests showed no statistically significant differences between the groups, we found that TDP‐43 levels were higher in LATE‐NC(+), relative to LATE‐NC(‐) cases [mean=626.7 (SD=557.4) vs. 449.6 (395.1) pg/mL]. Moreover, the mean concentration of TDP‐43 tended to increase with increased staging of LATE‐NC (Table 1 and Figure 1).

**Conclusion:**

Our results provide preliminary evidence that plasma TDP‐43 levels have the potential to be an in vivo biomarker for LATE‐NC either in isolation or combined with other potential measures. To address this, our future work will include assessing plasma TDP‐43 levels in combination with measures of inflammation and/or neurodegeneration.